# Chip Appearance Defect Recognition Based on Convolutional Neural Network

**DOI:** 10.3390/s21217076

**Published:** 2021-10-25

**Authors:** Jun Wang, Xiaomeng Zhou, Jingjing Wu

**Affiliations:** 1 School of Mechanical Technology, Wuxi Institute of Technology, Wuxi 214121, China; 2 Jiangsu Key Laboratory of Advanced Food Manufacturing Equipment Technology, Jiangnan University, Wuxi 214122, China; zhouxiaomeng@jiangnan.edu.cn (X.Z.); wjjlady720@jiangnan.edu.cn (J.W.)

**Keywords:** chip appearance defects, data cleaning, convolutional neural network, pattern recognition

## Abstract

To improve the recognition rate of chip appearance defects, an algorithm based on a convolution neural network is proposed to identify chip appearance defects of various shapes and features. Furthermore, to address the problems of long training time and low accuracy caused by redundant input samples, an automatic data sample cleaning algorithm based on prior knowledge is proposed to reduce training and classification time, as well as improve the recognition rate. First, defect positions are determined by performing image processing and region-of-interest extraction. Subsequently, interference samples between chip defects are analyzed for data cleaning. Finally, a chip appearance defect classification model based on a convolutional neural network is constructed. The experimental results show that the recognition miss detection rate of this algorithm is zero, and the accuracy rate exceeds 99.5%, thereby fulfilling industry requirements.

## 1. Introduction

Due to the advantages of being noncontact, nondestructive, full field, and of high precision, fringe projection profilometry (FPP) plays an important role in some academic and applied fields, such as product inspection, reverse engineering, and computer animation, [1,2,3,4,5]. Recently, with the development of high-speed imaging sensors and digital projection technology (e.g., the digital-light-processing module developed by Texas Instruments), it is possible to reach a higher level of quality and speed [6,7]. For this reason, researchers have started to expand the application domain of FPP to include, for example, biomechanics, on-line inspection, human-computer interaction, robot navigation, and solid mechanics [8].

Owing to the rapid development of information technology, electronic products are ubiquitous in various areas pertaining to the national economy and all aspects of society. Chips are basic carriers of electronic products. Because of equipment, environmental, and human factors, defects are inevitable during chip production. After a batch is completed, the chips are inspected visually to detect the appearance quality, something that pertains to surface detection research. Although general methods and theories exist for surface detection, owing to the significant differences in surface detection problems in different application fields, different methods have been developed for specific research fields, these include copper strip surface detection [1], gun barrel surface detection [2], fabric surface detection [3], asphalt surface detection [4], and crankshaft surface detection [5].

The development of surface detection technology has resulted in increased attention toward chip surface detection. Chiou et al. [6] detected defects including stains, scratches, solder masks, and pinholes in ball-grid-array-type printed circuit boards (PCBs), and classified the detected defects using a backpropagation neural network. Su et al. [7] demonstrated a nondestructive inspection method for the defect detection of flip chips using ultrasonic excitation and a laser scanning vibrometer. Tsai and Lin [8] proposed two entropy measures pertaining to chromatic and structural regularities for the surface inspection of gold fingers on PCBs, and various defects such as pinholes, copper exposure, nicks, and roughness were detected. Chang et al. [9] adopted a hybrid approach that combined a referential approach for case-based reasoning and a rule-based approach to construct an advanced PCB inspection system that can effectively detect defects in PCBs, e.g., open circuits, short circuits, indentation, and particles. Huang et al. [10] proposed an inspection algorithm composed of image segmentation and defect detection for detecting defects on PCB circuitries, e.g., broken circuits, short circuits, pinholes, over-etching, inadequate etching, and copper residue. Benedek [11] proposed a novel hierarchical marked point process framework for optical scooping analysis in PCBs and incorporated solder paste extraction and scooping error detection in a joint probabilistic approach. Wang et al. [12] presented a method for PCB welding spot detection using a series of image processing algorithms based on an automatic threshold segmentation algorithm and computer morphology. The methods above can effectively detect defects on a chip surface that are significantly smaller than the background area, and the defect features can be effectively extracted. Owing to weak defects on the chip surface caused by low contrast between the defects and background, as well as small defects, information regarding the defect target, background, noise, etc. is within a narrow gray scale range and difficult to distinguish, thereby complicating the automatic detection of chip surface defects.

Deep learning (DL) can approximate complex functions through multilayer networks and learn the essential features of data from many sample sets. Since it’s introduction by Hinton and Salakhutdinov [13] in 2006, DL has yielded significant achievements in computer vision, speech recognition, natural language processing, and other fields. Owing to the development of DL, surface detection methods based on DL have been actively investigated. Zhang et al. [4] proposed an efficient network architecture based on a convolutional neural network (CNN), named CrackNet, for the automated detection of pavement cracks on asphalt surfaces. Ren et al. [14] presented a generic DL-based automated surface inspection method including feature transfer from a pretrained DL network and the convolution of a patch classifier over an input image; the method demonstrated favorable detection capability on wood surface defects. Li et al. [3] proposed a Fisher-criterion-based stacked denoising autoencoder framework, by which fabric patches were efficiently classified into defect-free and defective categories. Mei et al. [15] proposed a novel approach known as multiscale convolutional denoising autoencoder, which had different Gaussian pyramid levels for detecting and localizing defects with defect-free samples for model training; the approach is effective for homogeneous and nonregular textured surfaces.

In summary, DL can extract and combine the underlying features of samples to identify hidden features, which are used widely in surface defect detection. Various types of chip appearance defects exist, and any background changes will cause random changes in the character and location of the defects. As such, a recognition method based on general feature extraction cannot identify defects effectively. Therefore, a chip appearance defect detection algorithm based on a CNN is proposed herein. The algorithm comprises primarily three procedures: image preprocessing, accurate location of the region of interest (ROI), and chip defect recognition.

The main contributions in this study are summarized as follows:

(1). An adaptive threshold segmentation algorithm based on light and shade coefficient is proposed to improve the classical OSTU method [16] used to address the problem of uneven ROI gray scales caused by pin frames, welding wire oxidation, and defects. The results show that the improved OSTU method is robust and accurate for the foreground of a large gray scale.
(2). A template-matching algorithm based on row and column statistical characteristics is proposed to solve random deformations in frames and welding wires, as well as the problem of epoxy resin with uneven distributions interfering with the extraction results of the chip plastic encapsulation area. The results confirm the robustness, short operating time, and high matching accuracy of the algorithm.
(3). An automatic data sample cleaning algorithm based on prior knowledge is proposed to fulfill the requirements of a large sample training network to ensure the accuracy of the sample set and the minimum number of dirty samples. The results show that data cleaning can effectively improve the reliability of the samples.

## 2. Principle

**Figure 1:**
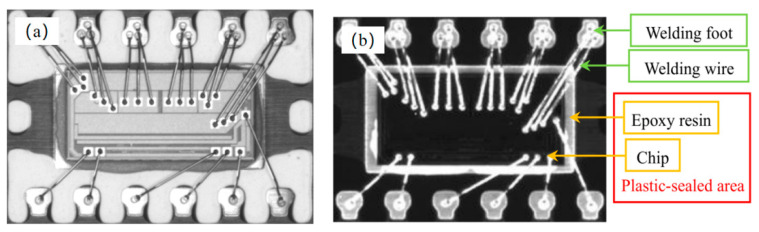
Image of chip. (**a**) Plastic package side; (**b**) pin side.

**Figure 2:**
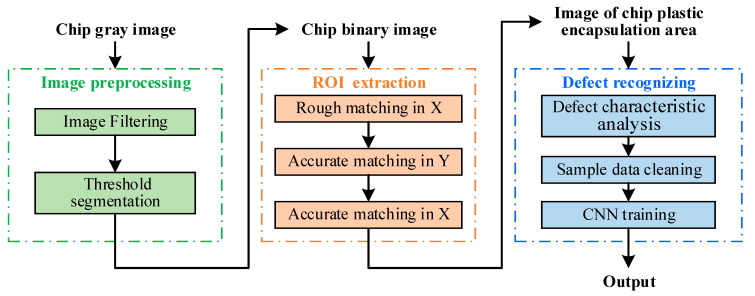
Overall scheme of chip surface defect-detection algorithm.

An image of the plastic encapsulation and pin sides of a chip is shown in Figure 1. The chip comprised four components: a welding foot, a welding wire, an epoxy resin, and a chip. The epoxy resin and chip constituted the enclosed area of the chip. This study focused on the surface defect detection of the enclosed area. The overall algorithm for chip appearance defect detection, as shown in Figure 2, comprised three procedures: (1) image preprocessing (see Section 2.1), (2) ROI extraction (see Section 2.2), and (3) defect recognition (see Section 2.3).

### 2.1. Image Preprocessing

The image preprocessing performed in this study included two procedures: (1) Image filtering and (2) threshold segmentation. Image filtering was performed to eliminate image noise, whereas threshold segmentation was performed to convert grayscale images into binary images.

#### 2.1.1. Image Filtering

The chip image noise was primarily caused by fine dust on the pin frame. Therefore, a median filter was adopted to de-noise the fine dust while maintaining the detailed features of the image as much as possible. The median filtering formula is expressed as follows:

(1) G(x,y)=med{F(x−k,y−l),(k,l∈W)},

where *F*(*x*, *y*) is the original image, *G*(*x*, *y*) the processed image, and *W* a two-dimensional template. In this study, *W* was a 5 × 5 kernel.

#### 2.1.2. Threshold Segmentation

**Figure 3:**
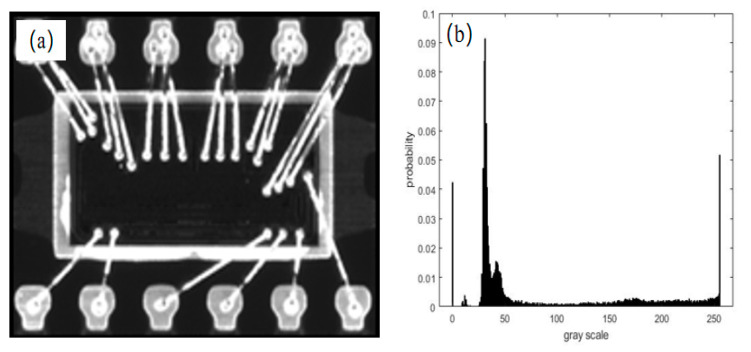

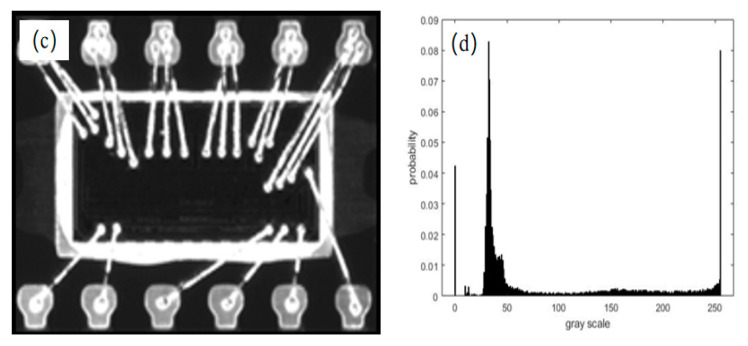
Pin side of chip image and its gray histogram. (**a**) Pin side in bright field; (**b**) gray histogram of pin side in bright field; (**c**) pin side in dark field; (**d**) gray histogram of pin side in dark field.

**Figure 4:**
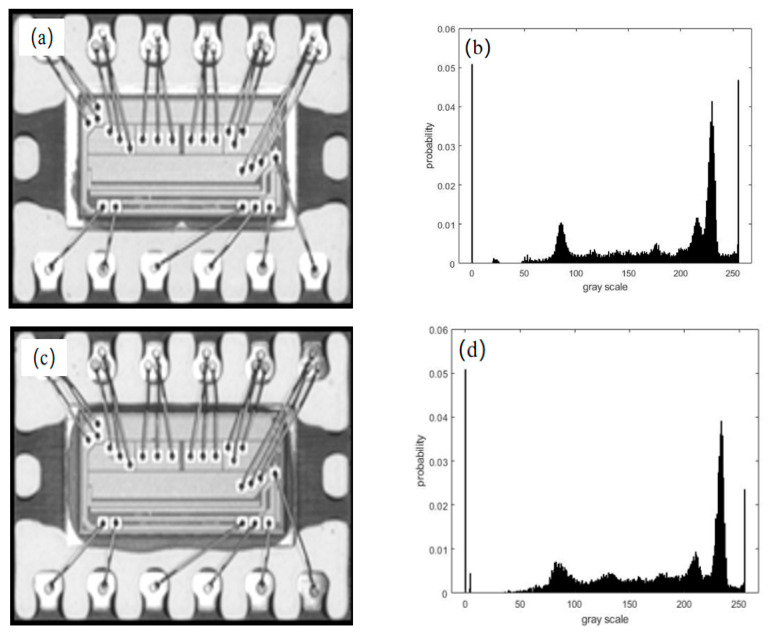
Plastic package side of chip image and its gray histogram. (**a**) Plastic package side in bright field; (**b**) gray histogram of plastic package side in bright field; (**c**) plastic package side in dark field; (**d**) gray histogram of plastic package side in dark field.

The solder oxidation of the pin frame and welding wire, as well as the uneven distribution of the epoxy and resin resulted in a gradient in the gray level in the bright/dark field of the pin side of the chip image. As shown in Figure 3, in the dark field, the gray histogram exhibited poor contrast, and the welding feet, expected as the foreground, indicated a lower gray level in the gray histogram owing to uneven oxidation. Conversely, in the bright field, the welding feet, welding wires, and pins as the foreground indicated high contrast to the background. Moreover, the gray distribution for the plastic package side of the chip image in the bright and dark fields was the same as that at the pin side, as shown in Figure 4. Therefore, the classic OSTU was improved in this study as follows: The bright and dark fields were assessed adaptively based on the average gray level of the gray image. An adaptive coefficient k was added to Equation (2) to improve the mean gray values of the welding pins, welding wires, and pins in the whole image, where k depends on the mean gray value of the entire image, represented by *u*′.

(2) $$\begin{array}{cc} \{\begin{array}{l} u’=w_{0}u_{0}+kw_{1}u_{1}\\ u=w_{0}u_{0}+w_{1}u_{1} \end{array} & k=\{\begin{array}{cc} 1 & u>50\\ \frac{50}{u} & u\leq 50 \end{array} \end{array}.$$

**Figure 5:**
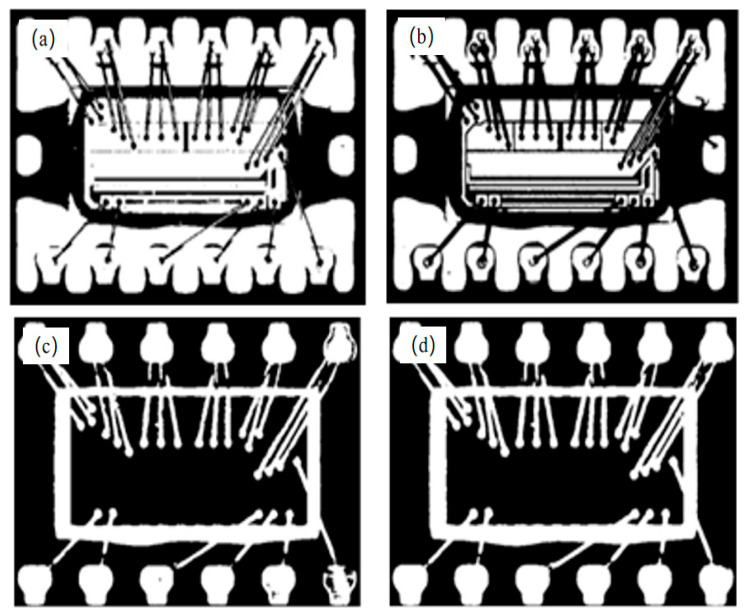
Comparison of thresholding segmentation effect between the classical OSTU method and the improved OSTU method for dark field images. Binary image of plastic package side obtained using (**a**) classical OSTU and (**b**) improved OSTU. Binary image of pin side obtained using (**c**) classical OSTU and (**d**) improved OSTU.

In the above, *w*_0_*u*_0_ and *w*_1_*u*_1_ are the probability/mean of the background and foreground, respectively. Figure 5 shows the corresponding binary images of the threshold segmentation by the classical and improved OSTU methods, and it can be concluded that the improved OSTU method can effectively distinguish the foreground and background in dark field images.

### 2.2. ROI Extraction

To extract the plastic-sealed region from the chip image, template-matching localization should be performed. The classic template-matching methods primarily include the mean absolute differences algorithm, sum of absolute differences algorithm (SAD), sum of squared differences (SSD), mean square differences (MSD), and normalized cross correlation algorithm (NCC). The SAD and SSD, which are based on pixels, have high complexity, and are easily disturbed by noise. Standard deviation is introduced into the NCC such that the global gray level in the image will not be easily disturbed. Therefore, an improved NCC was adopted in this study for template matching.

**Figure 6:**
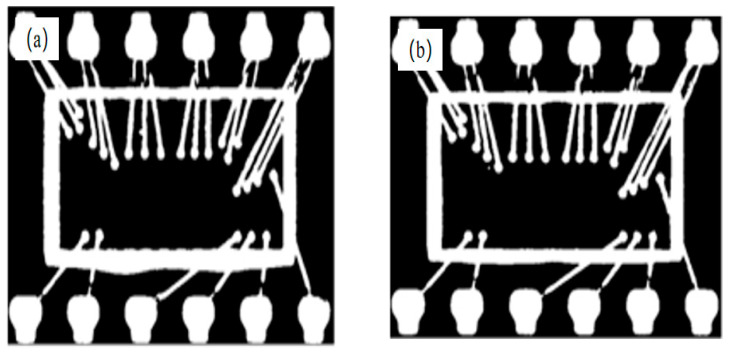
Binary image of pin side. (**a**) Unevenly and (**b**) evenly distributed epoxy resin.

**Figure 7:**
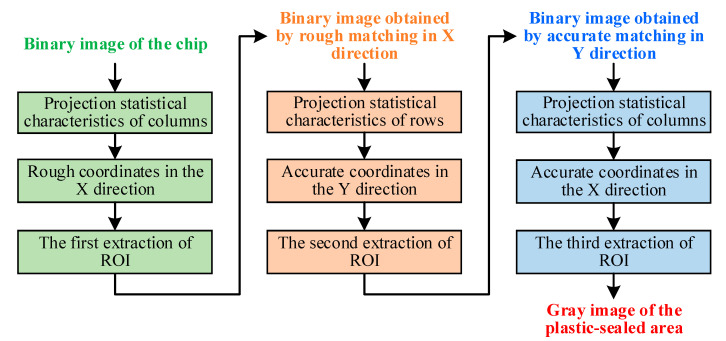
Flowchart of improved NCC template-matching algorithm for ROI extraction.

In some chips, the epoxy resin was unevenly distributed, as shown in Figure 6. The resulting white area after threshold segmentation was in contact with the chip; this hindered the template matching of the chip plastic-sealed region. Therefore, the projection statistical characteristics of columns and rows can be adopted for ROI extraction to not only overcome the significant amounts of computation in per-pixel feature extraction, but also to preserve the regional statistical features of the plastic-sealed area. A flowchart of the improved NCC template-matching algorithm for ROI extraction is shown in Figure 7.

**Figure 8:**
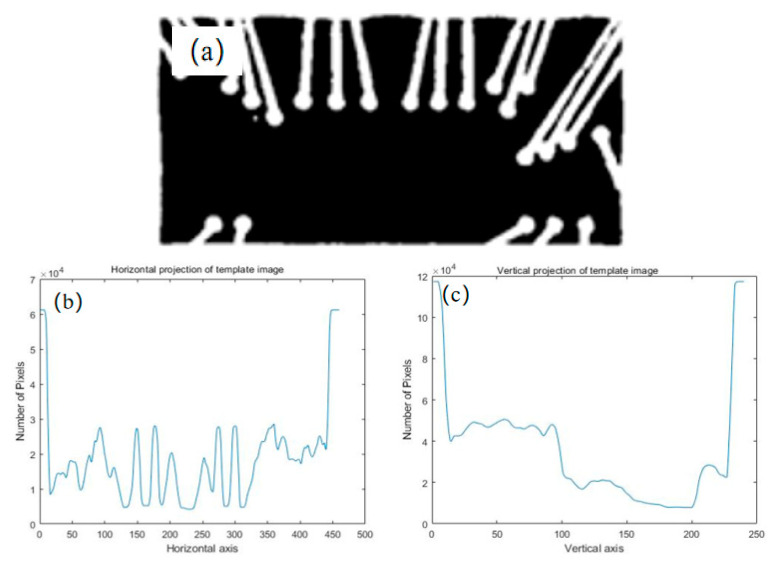
Binary image of template and its projection. (**a**) Binary image, (**b**) vertical projection, and (**c**) horizontal projection of template.

**Figure 9:**
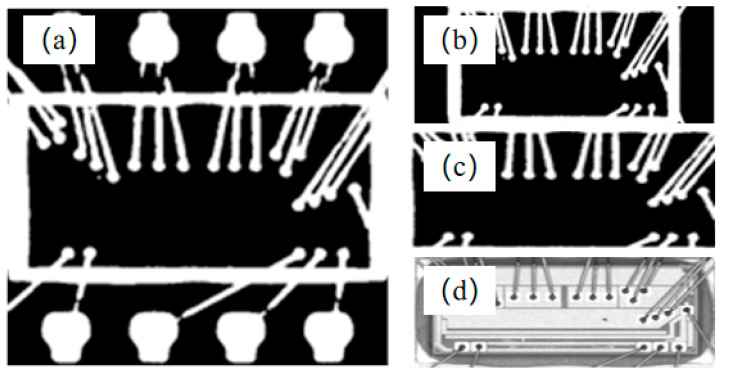
Images obtained during template matching using improved NCC. Binary image obtained via (**a**) approximate matching in X-direction and (**b**) accurate matching in Y-direction. (**c**) Binary image of plastic-sealed region of chip. (**d**) Gray image of plastic-sealed region of chip.

The mathematical description of the improved NCC template-matching algorithm based on the projection statistical features of the columns or rows is as follows:

(3) $$R(i)=\frac{\sum_{m=1}^{M}S_{i}(m)T_{i}(m)}{\sqrt{\sum_{m=1}^{M}{\left[S_{i}(m)\right]}^{2}}\sqrt{\sum_{m=1}^{M}{\left[T_{i}(m)\right]}^{2}}},$$

where *R* is the similarity metric, *T* the projection vector of the template of the plastic-sealed area (*M*), and S the projection vector of the binary image of the chip. In addition, the upper left corner of the image is the origin (0, 0), the horizontal axis is the *X*-axis, and the vertical axis is the *Y*-axis. Figure 8 shows a binary image of the template and its vertical and horizontal projections. Figure 9 shows the images obtained during the improved NCC template-matching process. It was clear that the improved NCC template-matching algorithm successfully extracted the plastic-sealed area of the chip.

**Figure 10:**
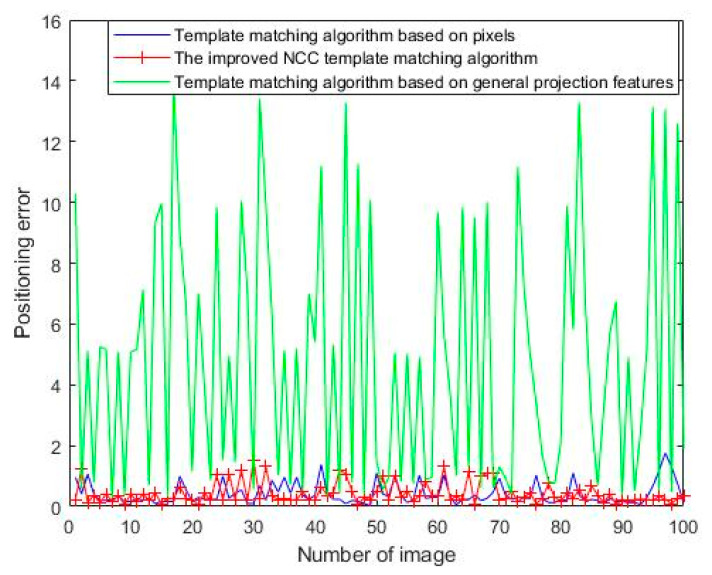
Positioning error comparison of three template-matching algorithms.

A comparison of three template-matching algorithms (i.e., (1) a template-matching algorithm based on pixels, (2) a template-matching algorithm based on general projection features, and (3) the improved NCC template-matching algorithm) was performed on 100 images to verify the efficiency and accuracy of the proposed algorithm. Comparisons of the operating time and positioning error are shown in Table 1 and Figure 10, respectively. In this study, the positioning error is described as the Euclidean distance between the matching position and the actual position.

(4) $$\delta =\sqrt{{\left(x-x_{0}\right)}^{2}+{\left(y-y_{0}\right)}^{2}},$$

where (*x*, *y*) is the matching position, and (*x*_0_, *y*_0_) is the actual position. The positioning error of the template-matching algorithm based on general projection features was significant and hence did not fulfill the accuracy requirement. The operating time of the template-matching algorithm based on pixels exceeded 100 ms, which did not satisfy the cycle time requirement. The operating time of the improved NCC template-matching algorithm proposed herein was less than 30 ms, and the positioning error was small, which can satisfy the actual demand.

### 2.3. ROI Extraction

**Figure 11:**
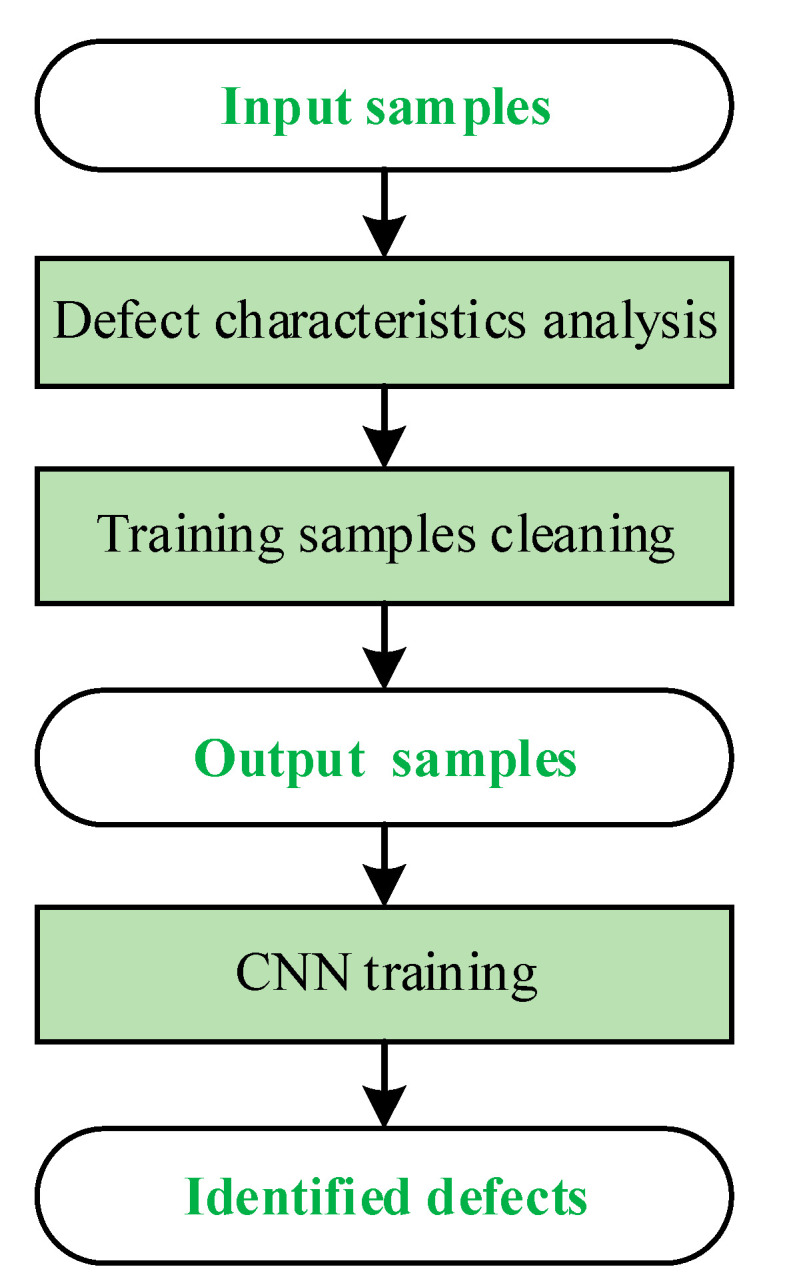
Flowchart of defect recognizing algorithm.

The defect characteristics of the chips were analyzed. Defect features with significant and insignificant intra-class variances in each sample were analyzed to identify similar features among different defect types such that interference features that can easily cause misidentification can be eliminated. After performing data cleaning on the training samples, the AlexNet model was used to train the CNN. A flowchart of the defect recognition algorithm is shown in Figure 11.

#### 2.3.1. ROI Extraction

**Figure 12:**
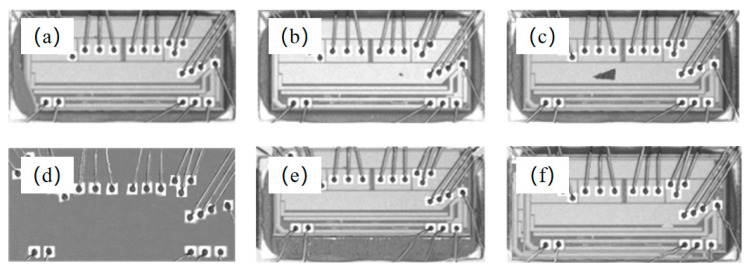
Six types of chip surface defects: (**a**) Edge defect; (**b**) foreign-body defect; (**c**) fragmentation defect; (**d**) void chip defect; (**e**) incorrect position defect; (**f**) repeatedly pasting defect.

After the ROI of the chip image was extracted, the defect characteristics of the chip were analyzed and categorized into six types: edge defects, foreign-body defects, fragmentation defects, void chip defects, incorrect position defects, and repeatedly pasting defects. Details pertaining to these defects are described as follows:

Edge defect: Chips are generated by cutting round wafers into small pieces. The chip cut from the circular edge of the wafer has an irregular shape, which does not satisfy the process requirements (see Figure 12a).

Foreign-body defect: During chip production, the chip is transmitted after being drawn by a vacuum nozzle; therefore, oil from the vacuum nozzle will adhere to the chip surface, thereby rendering the surface of the chip sticky. As such, particles such as dust can be trapped on the chip. Typically, foreign bodies discovered on the surface of chips are dust from the environment or randomly shaped broken wires caused by machine failure. The characteristics of the dust and broken wires differ significantly from those of the chip (see Figure 12b).

Fragmentation defect: When the chip is drawn by the vacuum nozzle, the excessive nozzle pressure generated causes chip fragmentation. The defect area is typically located near the center of the chip. Because the suction nozzle and the plastic sealing surface are primarily inclined contacts, the shape of the fracture defect is typically a strip or a block (see Figure 12c).

Void chip defect: The chip is drawn away by the vacuum nozzle, or the chip is not pasted, thereby resulting in a chip with only welding feet and welding wires (see Figure 12d).

Incorrect position defect: The chip is not pasted in the correct location (see Figure 12e).

Repeatedly pasting defect: The vacuum nozzle did not perform as intended when absorbing the chip, resulting in multiple chip pasting (see Figure 12f).

**Figure 13:**
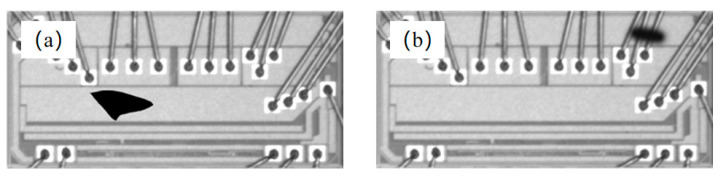
Comparison of foreign-body and fragmentation defects. (**a**) Foreign-body defect; (**b**) fragmentation defect.

As shown by the chip surface defect images presented in Figure 12, it is clear that edge, void chip, incorrect position, and repeatedly pasting defects differ significantly and are hence easily distinguishable. Conversely, the difference between foreign-body and fragmentation defects is ambiguous; therefore, they can be easily misidentified, as illustrated in Figure 13. In addition, because the grayscale of foreign-body defects is similar to that of fragmentation defects, it is difficult to distinguish the two defects using grayscale features. In terms of geometric features, the geometrical sizes of these two defects are random; therefore, they cannot be distinguished by area and perimeter. The analysis shows that the chip fragmentation is primarily caused by excessive force of the vacuum nozzle when drawing the chip, i.e., the geometric center of the fragmentation defect is primarily located at the center of the chip. However, foreign-body defects are primarily caused by dust or falling welding wires, and the geometric center of foreign-body defects on the chip is random. Therefore, fragmentation and foreign-body defects cannot be detected based on the geometric center position.

Meanwhile, when the foreign body is dust, the defect area is round. In this regard, Equation (5) is introduced, where the compactness *C* can be used to distinguish the two types of defects, *S* is the area of the defect area (i.e., the number of pixels in the defect area), and *L* is the boundary length of the defect area (i.e., the number of edge pixels). When the foreign body was welded, the defect area resembled a rectangle. In this case, Equation (6) was adopted, where *Rq* represents the ratio between *S* and the minimum bounding rectangle; *L_s_* and *L_l_* denote the short and long sides, respectively.

(5) $$C=\frac{4\pi S}{L^{2}},$$

(6) $$R_{q}=\frac{S}{L_{s}L_{l}},$$

#### 2.3.2. Cleaning Algorithm for Training Samples

**Figure 14:**
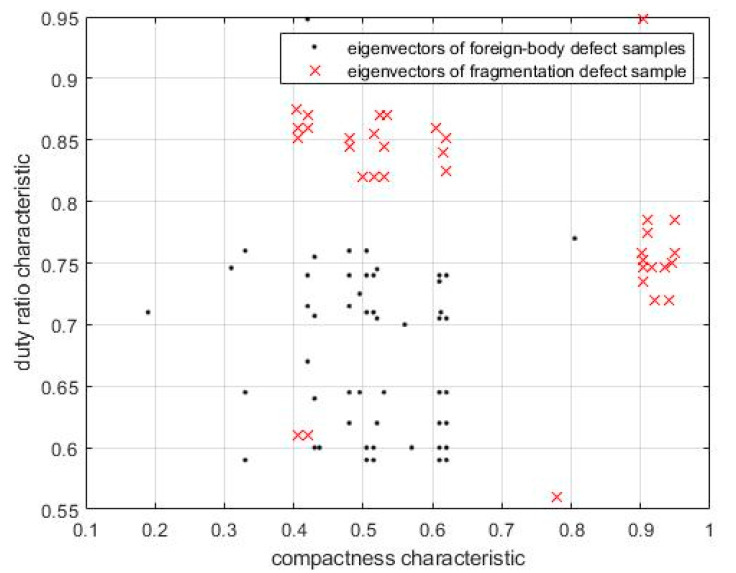
Eigenvector distribution of foreign-body defect and fragmentation-defect samples.

The eigenvector of a foreign-body defect (Equation (7)) and fragmentation defect (Equation (8)) can be expressed as follows:

(7) $$A\{(x_{A0},y_{A0}),(x_{A1},y_{A1}),\cdots (x_{AN-1},y_{AN-1})\},$$

(8) $$B\{(x_{B0},y_{B0}),(x_{B1},y_{B1}),\cdots (x_{BN-1},y_{BN-1})\},$$

where *x_Ai_* is the compactness of the defect area, *y_Ai_* the duty ratio characteristic of the defect area, *x_Bi_* the compactness of the defect area, *y_Bi_* the duty ratio of the defect area, and *N* the number of training samples. The eigenvector distributions of the foreign-body defect and fragmentation defect samples are presented in Figure 14. The following two phenomena were observed: (1) the eigenvector distribution of the foreign-body defect samples was relatively concentrated, and (2) most of the samples were concentrated in areas where the *X*-axis coordinate (denoting compactness) exceeded 0.9, or the *Y*-axis coordinate (denoting duty ratio) exceeded 0.8. By contrast, the eigenvector distribution of the fragmentation defect samples was relatively scattered, primarily in the region where the *X*-axis coordinate was less than 0.7, and the *Y*-axis coordinate was less than 0.8.

To simplify computation and reduce interference between compactness and duty ratio, using the coordinate value as the metric, we set the threshold value *D* (0.8 in this study) to screen foreign-body defect samples whose *X*- or *Y*-axis coordinate exceeded or were equal to *D*, and fragmentation defect samples whose X- and *Y*-axis coordinates were both less than *D*. The formula pertaining to the sample screening is as follows:

(9) $$\{\begin{array}{cc} if(x_{Ai}<\Delta D)and(y_{Ai}<\Delta D) & delete(A_{i})\\ else & continue \end{array},$$

(10) $$\{\begin{array}{cc} if(x_{Bi}<\Delta D)or(y_{Bi}<\Delta D) & delete(B_{i})\\ else & continue \end{array},$$

#### 2.3.3. CNN Training

**Figure 15:**
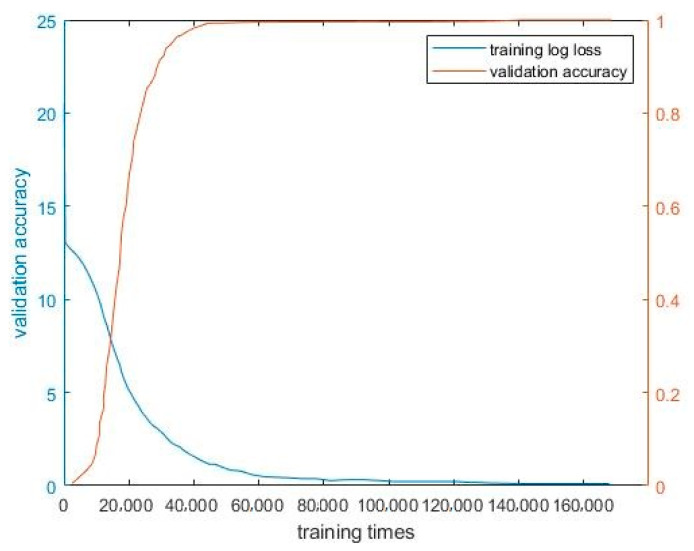
Loss function and recognition accuracy with respect to training time.

In this study, 1000 chip images of each defect type and 1000 chip images without defects were selected. Therefore, 7000 images were used as the sample database, 70% of which was used as the training sample set, and 30% as the test sample set. After cleaning the training dataset using the method described in the previous section, the AlexNet model was used for training. The training sample set was trained 160,000 times, the loss function was output once every 1000 times, and a recognition accuracy test was performed once every 2000 times. As shown in Figure 15, as the training time increased, the recognition accuracy first increased significantly, then stabilized, and finally reached 99.73%. By contrast, the loss function declined significantly at first and then stabilized to 0.17%.

The test sample set was input to the trained model for testing, and the chip defect detection results are listed in Table 2. The columns represent the actual defect types, whereas the rows represent the defect types that were automatically identified by the CNN model, as described below. As shown in the table, the result with defects (from II to VII) are not confounded into no-defect result (I), that is, the missed detection rate of the test samples was 0. In addition, only a few foreign-body and fragmentation defects were mistakenly detected, and the test accuracy reached 99.76%.

## 3. Result and Discussion

The proposed algorithm was implemented in the Windows 10 operating system and coded using LabVIEW and Visual C++. Real-time performance and accuracy were tested on a personal computer equipped with an Intel(R) Core(TM) i7-7700HQ, 16 GB of memory, and 256 GB of storage on a solid-state drive.

### 3.1. Real-Time Performance

The data cleaning and training processes did not constitute the online defect detection as they were performed during offline training. In other words, those processes were not included in the real-time performance test. For the real-time test, 1000 images that resembled Figure 1a or Figure 1b were used for the method proposed herein, and the operating times of the main stages were 65.32 (threshold segmentation), 12.91 (position) and 130.76 ms (defect detection). The total time of the three main stages was approximately 221.10 ms, where defect detection consumed the longest time. In terms of operating time, the algorithm presented herein demonstrated decent performance with satisfactory accuracy.

### 3.2. Accuracy Performance

For the accuracy performance test, 1000 images were used. These images show defective and non-defective chips. The chip defects included edge, foreign-body, fragmentation, missing, misplace, and number defects. After image preprocessing, the proposed method was used for classification, and the results are listed in Table 3. The test results show 10 images presenting both foreign-body and fragmentation defects, five images presenting only foreign-body defects, and five images presenting fragmentation defects. Moreover, except for the two abovementioned defects, the other defects were insignificant. Therefore, the accuracy rate was 99.56%. Meanwhile, we listed the accuracy results of several related methods, as listed in Table 3. It can be seen from the comparison that our proposed method has the highest accuracy, which satisfies practical requirements.

As shown in Table 4, misdetection occurred in the defect classification. This is attributable to two main reasons: (1) Image preprocessing was not sufficiently meticulous, i.e., interference information was retained. (2) The images of the foreign-body and fragmentation defects were similar, which can easily interfere with the classification. In addition, the testing data were directly obtained from the actual scene without cleaning, which may have affected the accuracy.

## 4. Conclusions

Herein, a novel chip appearance defect recognition algorithm based on a CNN was proposed. The algorithm exhibited the following characteristics:

(1). In segmenting images with non-uniform bright and dark fields, our proposed OTSU method demonstrated significant advantages over the traditional OTSU method.
(2). Our proposed localization algorithm offered high efficiency, high accuracy, robustness, and high practicality.
(3). Effective data cleaning was performed prior to classification, thereby improving the recognition rate of the system.

## Figures and Tables

**Table 1:** Operating time comparison of three template-matching algorithms.

Template-Matching Algorithm	Average Running Time
Template-matching algorithm based on pixels	105 ms
Improved NCC template-matching algorithm	21 ms
Template-matching algorithm based on general projection features	16 ms

**Table 2:** Test results based on AlexNet model after training. I: no defect; II: edge defect; III: foreign-body defect; IV: fragmentation defect; V: void chip defect; VI: incorrect position defect; VII: repeatedly pasting defect.

RESULT	I	II	III	IV	V	VI	VII
I	300						
II		298		3			
III			300				
IV		2		297			
V					300		
VI						300	
VII							300

**Table 3:** Accuracy comparison results of different methods.

Method	Lin’s [17]	Redmon’s [18]	Chen’s [19]	Fu’s [20]	Our Proposed
Accuracy Rate	61.59%	80.30%	95.28%	88.40%	99.56%

**Table 4:** Statistics of chip plastic surface defect image recognition results. I: no defect; II: edge defect; III: foreign-body defect; IV: fragmentation defect; V: void chip defect; VI: incorrect position defect; VII: repeatedly pasting defect.

Defect	I	II	III	IV	V	VI	VII
I	990						
II		1000					
III	3		985	18			
IV	7		15	982			
V					1000		
VI						1000	
VII							1000
Accuracy Rate	0.99	1	0.985	0.982	1	1	1

## Data Availability

The datasets used and/or analyzed during the current study are available from the corresponding author on reasonable request.
